# Bioaccessibility and risk assessment of heavy metals, and analysis of arsenic speciation in *Cordyceps sinensis*

**DOI:** 10.1186/s13020-018-0196-7

**Published:** 2018-07-31

**Authors:** Li Zhou, Sheng Wang, Qingxiu Hao, Liping Kang, Chuanzhi Kang, Jian Yang, Wanzhen Yang, Jingyi Jiang, Lu-Qi Huang, Lanping Guo

**Affiliations:** 1 National Resource Center for Chinese Materia Medica, State Key Laboratory Breeding Base of Dao-di Herbs, China Academy of Chinese Medical Sciences, Beijing, 100700 People’s Republic of China; 2 College of Traditional Chinese Medicine, Guangdong Pharmaceutical University, Guangdong, 510006 China

**Keywords:** *Cordyceps sinensis*, Heavy metals, As, Gastrointestinal digestion, Risk assessment

## Abstract

**Background:**

*Cordyceps sinensis* (*C. sinensis*) is a famous and precious Traditional Chinese Medicine (TCM), while frequent reports of heavy metals, especially arsenic, exceeding standards in *C. sinensis* in recent years have raised concerns of its safety. Therefore, it is urgent for a research on heavy metals (Cu, Pb, As, Cd, Hg) in *C. sinensis*, of its bioaccessibility, dietary exposure estimation, arsenic speciation analysis and health risks assessment to human body.

**Methods:**

Three 30 g batches of mixed wild growth *C. sinensis* samples were collected from Qinghai Province and each batch were divided into three parts: the whole *C. sinensis*, the stroma and the caterpillar body. The in vitro gastrointestinal method was used to evaluate the bioaccessibility of the heavy metals in the samples. The arsenic speciation analysis in the in vitro gastrointestinal solutions and dilute nitric acid extracted solutions were conducted using high performance liquid chromatography–inductively coupled plasma mass (HPLC–ICP-MS) method. Finally, the target hazard quotient (THQ) developed by the US EPA (1989) was used to assess the health risks of heavy metals in *C. sinensis*.

**Results:**

The contents of Cu, Pb, Cd and Hg in the stroma were higher than those in the caterpillar body. In contrast, As was mainly found in the caterpillar body. In the whole *C. sinensis*, the average bio-accessibilities of Cu, Pb, As, Hg and Cd were 41.29, 40.11, 64.46, 18.91, and 81.14%, respectively. While in the caterpillar body, the corresponding bio-accessibilities values were 48.26, 42.92, 66.15, 12.86, 87.07%, respectively, and were 38.30, 30.53, 30.18, 7.46, and 82.30%, respectively in the stroma part. Different arsenic speciations of arsenite [As(III)], arsenate [As(V)] and trace amounts of methylarsonic acid [MMA] were detected. Of the total As, 8.69% was in inorganic form, which was also the major form of dissolved As. Among the extracted inorganic species, the concentrations of As(III) and As(V) were 0.56 ± 0.16 and 0.29 ± 0.06 mg kg^−1^, respectively. In the gastrointestinal solutions, only As(III) and As(V) could be detected; the sum content of the two species was 2.00–2.73%. The bioaccessibility target hazard quotient (BTHQ) values for Cu, Pb, As, Cd and Hg in *C. sinensis* were 0.0041, 0.0040, 0.5334, 0.0020 and 0.0005, respectively, all less than 1.

**Conclusion:**

None of the five heavy metals in *C. sinensis* can be 100% absorbed by human body. The content of arsenic in *C. sinensis* is high, but the strong toxic inorganic arsenic accounted for only 8.69%. The heavy metals in *C. sinensis* presented no obvious risks to human health in a reasonable taking way.
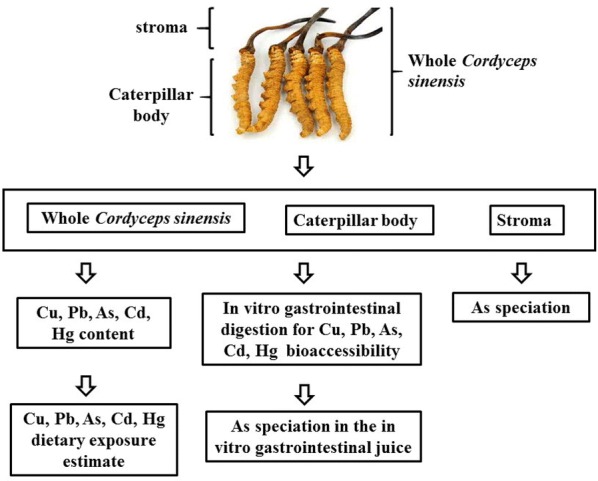

**Electronic supplementary material:**

The online version of this article (10.1186/s13020-018-0196-7) contains supplementary material, which is available to authorized users.

## Background

With the rapid development of modern industry, a large number of heavy metals are discharged into the environment along with waste water, waste residue and waste gas from industrial and agricultural activities, resulting in environmental heavy metal contamination [1]. Heavy metals in the natural environment could enter human body via a variety of ways and can accumulate in the body [2–4], affecting normal organism physiological functions and causing inflammation and a variety of diseases, including cancer [5].

*Cordyceps sinensis* (BerK). Sacc is a parasitic fungus that grows on the larva of Lepidoptera. It is a Traditional Chinese Medicine known as DongChong-XiaCao (winter worm-summer grass) [6, 7]. It can effectively regulate the immune system and protect the nervous system, with anti-tumour properties [7, 8]. In recent years, the price of *C. sinensis* has risen dramatically [9], due to its efficacy and the scarcity of resources, and at present, the price of *C. sinensis* in different specifications ranges from $30,000 to $100,000 per kilogram. At the same time, concerns about the heavy metal As in *C. sinensis* have also grown with reports of high As content in *C. sinensis*. Our previous study found that contents of arsenic in 20 batches of *C. sinensis* were 2.59–12.56 mg kg^−1^ [10]. The excess rate of As was 88.24% based on the international standards of Chinese Medicine-Chinese Herbal Medicine Heavy Metal Limit (4 mg kg^−1^ for As) [11].

Chemical speciations of arsenic in nature include arsenate [As(V)], arsenite [As(III)], methylarsonic acid (MMA), dimethylarsinic acid (DMA), arsenobetaine (AsB), and arsenocholine (AsC), etc. [12]. Studies have shown that inorganic arsenic species are the most dangerous species of arsenic, and As(III) is more toxic than As(V), with the toxicity of As(III) almost 60 times that of As(V). MMA and DMA are considered to be hypotoxic; however, arsenosugars and arsenic greases are believed to be non-toxic [13, 14]. As(III) has a strong affinity with enzymes and the SH and NH groups of proteins in the body, and it can combine with thiols to form stable complexes that can hinder the cell’s normal metabolism and inhibit some enzymes [15, 16].

At present, the heavy metal problem of *C. sinensis* has received extensive attention. Previous researches on the heavy metal of *C. sinensis* mainly focused on its determination method and content, while researches on the species of As contained and the bioaccessibilities and health risk of heavy metals of *C. sinensis* considering its usage were rarely reported. In this study, we focused on the dietary exposure and bioaccessibilities of As, Cu, Pb, Cd and Hg and the variety of As species present in *C. sinensis* [17, 18], aiming to assess the risks of heavy metals and supplement the research on the safety of *C. sinensis*.

## Methods

The Minimum Standards of Reporting Checklist contain details of the experimental design, and statistics, and resources used in this study (Additional file 1).

## Instrumentation

An ICP-MS (ICAP-Q, Thermo Fisher Scientific, Analytical System, United States, Massachusetts) operating under normal multi-element tuning conditions was used to determine the total levels of As, Cu, Pb, Cd and Hg. The main analytical parameters were as follows: RF forward power, 1550 W; plasma argon flow rate, 13.98 L min^−1^; auxiliary argon flow rate, 0.80 L min^−1^; nebulizer argon flow rate, 1.10 L min^−1^; integration time, 0.02 s per point; and points per peak, 3.

An ICP-MS was used as the detector system following species separation by HPLC for arsenic speciation analysis, and the column effluent was directly introduced into a nebulizer and spray chamber. Data were collected by single ion monitoring at m/z 75. For chromatographic separation, a high-pressure pump (Ultimate 3000 RSLCnano, Thermo Fisher Scientific, United States, Massachusetts) was used as the sample delivery system. A Dionex IonPacTM AS7 column (2 × 250 mm Thermo Fisher Scientific, United States, Massachusetts) was used for arsenic speciation analysis. The mobile phases were 0.1 mol L^−1^ ammonium carbonate (A) and 4 nmol L^−1^ ammonium carbonate (B). The elution gradient system was 0% B (0–2.5 min), 0–100% B (2.5–8 min), 100% B (8–10 min), 100–0% B (10–10.01 min), 0% B (10.01–20 min). The flow rate was 0.25 mL min^−1^. The injection volume was 20 μL.

### Calibration standards and reagents

The single-element standard solutions (1000 mg L^−1^) of Cu, Pb, As, Cd, and Hg, purchased from the National Standard Material Center (Beijing, China), were used as the standard calibration solutions. Standard solutions of As As(III), As(V), MMA, AsC and AsB, purchased from the National Standard Material Center (Beijing, China) were used as the standard source solutions, with their concentrations guaranteed. DMA certified reference material was prepared from commercially available reagents, with its purity evaluated before use. Working mixture standard solutions were prepared daily by diluting the source standard solutions to proper concentrations with pure water. Deionized water (18.2 MΩ) purified with a Milli-Q system (Millipore, Inc, Spain) was used for all solution preparation. Plasticware and glassware were maintained in 15% HNO_3_ for 24 h before use.

### Simulated gastrointestinal juices

Simulated gastric juice (pH 1.8 ± 0.1) was prepared by dissolving 10.0 g of pepsin in 800 mL deionized water, then 16.4 mL of dilute hydrochloric acid (prepared by diluting 234 mL of concentrated hydrochloric acid to 1000 mL with deionized water) was added, and finally, additional deionized water was added to bring the total volume up to 1000 mL.

Simulated intestinal juice was prepared just before use by mixing equal volumes of A and B solutions. (Solution A was 6.8 g of potassium dihydrogen phosphate dissolved in 500 mL of water, and 0.1 mol L^−1^ NaOH was used to adjust the pH to 6.8. Solution B was 10 g of pancreatin dissolved in water up to 500 mL) [19].

### *Cordyceps sinensis* samples

Three 30 g batches of *C. sinensis* samples were collected from Qinghai Province and were numbered 1, 2, and 3. All samples were dried at 40 °C and stored at 4 °C before analysis [20].

### Sample preparation for contents determination of Cu, Pb, As, Cd, and Hg by ICP-MS

Each batch of *C. sinensis* samples were equally divided into two parts. One part, the whole *C. sinensis* (W), was ground with a mortar and passed through a 50-mesh sieve and numbered W_1_, W_2_, and W_3_, respectively. The other part was separated into stroma (S) and caterpillar body (C), and those two portions were separately ground with a mortar and passed through a 50-mesh sieve and numbered S_1_, S_2_, S_3_ and C_1_, C_2_, C_3_, respectively.

The whole *C. sinensis* samples, stroma samples and caterpillar body samples were digested with a microwave digestion system. Approximately 0.2 g of each sample was precisely weighed and transferred in PTEF vessels with 5 mL of concentrated HNO_3_ and 1 mL of H_2_O_2_. The operating procedure of the microwave system was as follows: the samples were heated at 100 °C for 3 min in the 1st step, 160 °C for 8 min in the 2nd step, and 190 °C for 30 min in the 3rd step, and then cooled to room temperature. The sample solutions were diluted to 50 mL with pure water. The blank and reference materials (carrots) were prepared at the same time under the same procedure.

#### Method validation

The method was fully validated by characteristic indices including linearity, limit of detection (LOD), limit of quantitation (LOQ), accuracy, and stability.

### Extraction procedures for arsenic speciation analysis in the whole *C. sinensis*, stroma and caterpillar body by HPLC–ICP-MS

#### Dilute nitric acid solution extraction

5 mL of dilute nitric acid solution (HNO_3_: water = 2: 98) was added to precisely weighed 0.2 g samples of whole *C. sinensis*, stroma and caterpillar body, respectively. The mixtures were soaked overnight, then heated in a thermostatic water bath for 120 min at 90 °C and shaken for 1 min every 30 min. The mixture was cooled to room temperature and centrifuged at 11.5*g* for 10 min after extraction. The supernatants were removed, and 5 mL of dilute nitric acid solution was added to the remaining pellets. The extraction was repeated using procedure described above. The combined supernatants from the two rounds of extraction were analysed immediately after filtration. The procedure was performed in triplicate [21].

### In vitro gastrointestinal digestion of whole *C. sinensis*, stroma and caterpillar body for arsenic speciation analysis

#### Gastric digestion

The prepared gastric juice (5 mL) was added to 0.5 g of precisely weighted whole *C. sinensis*, stroma and caterpillar body samples, respectively. Then the samples were heated for 1 h in a water bath, shaken for 30 min in a shaker (250 r min^−1^), and heated for an additional 3 h at 38 °C. The mixture was centrifuged at 11.5*g* for 10 min, and the collected supernatant was used as gastric digestion extract solution.

#### Intestinal digestion

The prepared intestinal juice (5 mL) was added to the solid fraction obtained in the gastric digestion, following the digestion procedure as above. The residue was discarded.

The supernatant was passed through a 0.45 µm PVDF syringe-type filter before analysis. The procedure was performed in triplicate [22].

### Preparation of in vitro gastrointestinal solution samples for determination of Cu, Pb, As, Cd and Hg content by ICP-MS

After digestion, the two liquid extracts were mineralized in a microwave oven. The samples were appropriately diluted before analysis.

#### Health risk assessment of heavy metals

To evaluate the potential acute or long-term hazards from exposure to heavy metals in *C. sinensis*, the DI, DE, and THQ of heavy metals were calculated using the following formulas [23].

$$ {\text{DI}} = C \times {\text{ FIR}} $$

where DI is the dietary intake of the heavy metal (µg person^−1^ day^−1^), C is the measured concentration of the heavy metal in the samples and FIR is the daily dose (g), which was taken as the maximum daily dose (9 g) recommended by the “Chinese Pharmacopoeia” standard.

$$ {\text{DE }} = {\text{ DI}}/{\text{ WAB}} $$

where DE is the dietary exposure (µg kg^−1^ body weight day^−1^) and WAB is the average individual body weight (62 kg).

For noncancerous effects, the target hazard quotient (THQ) developed by the US EPA (1989) has been used to evaluate potential health risks associated with long-term exposure to chemical pollutants in foodstuffs and was therefore used to assess the health risks from heavy metals in *C. sinensis* over a lifetime in this study. A THQ value higher than 1 indicates that those ingested *C. sinensis* are affecting health adversely to some extent. And if the THQ value is less than 1, it suggests that the amount of heavy metal exposed to the body has no significant effect on human health.

$$ THQ = \frac{{C \times {\text{FE}} \times {\text{ED}} \times {\text{FIR}}}}{{{\text{WAB}} \times {\text{AT}} \times {\text{RFD}} \times 1000}} $$

$$ {\text{BTHQ }} = THQ \times {\text{Bio}}-{\text{accessibility}}_{\text{HM}} $$

where C is the measured concentration of heavy metal in *C. sinensis*; EF is the exposure frequency (90 day per year); ED is the exposure duration (50 years); FIR is the daily dose (9 g); AT is the averaging time for non-carcinogens (70 × 365 day); RFD is the oral reference dose provided by the US EPA (Cu = 0.04 µg g^−1^ day^−1^ body weight, Pb = 0.0035 µg g^−1^ day^−1^ body weight, As = 0.0003 µg g^−1^ day^−1^ body weight, Cd = 0.001 µg g^−1^ day^−1^ body weight, Hg = 0.0005 µg g^−1^ day^−1^ body weight); 10^−3^ is the unit conversion factor; WAB is the average individual body weight (62 kg).

It has been observed that using the total concentrations of heavy metals may overestimate the amount absorbed through oral ingestion; the oral bioaccessibility is defined as the fraction of a compound that is released from its matrix in the gastrointestinal tract, and thus becomes available for intestinal absorption, i.e., enters the blood stream [24]. The bioaccessibility value is used to avoid overestimating risk.

## Results

### Method validation for quantitative analysis of Cu, Pb, As, Cd and Hg

Performance parameters of the analytical method were evaluated, and the detection limits (DL) for the elements (expressed in µg L^−1^) were as follows: Cu: 0.007; Pb: 0.026; As: 0.010; Cd: 0.005; and Hg: 0.006. The quantification limits (QL) for the elements (expressed in µg L^−1^) were Cu: 0.020; Pb: 0.100; As: 0.030; Cd: 0.020; and Hg: 0.020. The standard curve prepared for each element was linear. The relative standard deviations (RSDs) were less than 3% (Table 1). The results of the reference material were in good consistence with the certified values (Table 2).

**Table 1:** Regression equations, correlation coefficients, linear ranges and detection limits

Analyte	Linear equation	*R* ^2^	Linear range (µg L^−1^)	LOD (µg L^−1^)	LOQ (µg L^−1^)
Cu	Y = 38371.1x + 1119.4	0.9999	0.02–200	0.007	0.020
Pb	Y = 230129.3x + 32337.6	0.9996	0.10–20	0.026	0.100
As	Y = 2504.2x + 32.7	0.9999	0.02–20	0.010	0.030
Cd	Y = 30243.4x + 300.6	0.9998	0.02–20	0.005	0.020
Hg	Y = 34165.1x + 84.7	0.9973	0.02–5	0.006	0.020

**Table 2:** The content of heavy metals in standard reference material for carrot

Analyte	Carrot (reference material)	
	Reference value (mg kg^−1^)	Measured value (mg kg^−1^)
Cu	4.1 ± 0.3	3.89
Pb	0.43 ± 0.07	0.39
As	0.11 ± 0.02	0.11
Cd	0.034 ± 0.004	0.036
Hg	0.0032 ± 0.0008	0.0026

### The bioaccessibility of heavy metals in different parts of *C. sinensis*

The average bioaccessibilities of Cu, Pb, As, Hg and Cd were 41.29, 40.11, 64.46, 18.91, and 81.14% in whole *C. sinensis*, respectively; 48.26, 42.92, 66.15, 12.86, and 87.07% in caterpillar body; and 38.30, 30.53, 30.18, 7.46, and 82.30% in stroma (Table 3). The bioaccessibilities of Cu, As, Hg and Cd in each part in the gastric extract were higher than those in the intestinal extract, and the vast majority of Cu and Cd were dissolved by the gastric juice. While the bioaccessibility of Pb in gastric juice was lower than that in intestinal juice. The dissolved quantity of each heavy metal is proportional to its total content of the sample. However, the bioaccessibilities of different heavy metals sometimes varies based on their binding forms and properties. The average contents of Cu, Pb, As, Hg and Cd were 15.02, 1.39, 13.47, 0.06, and 0.08 mg kg^−1^ in caterpillar body and 18.63, 2.06, 1.49, 0.07, and 0.22 mg kg^−1^ in stroma, respectively. The contents of Cu, Pb, Hg, and Cd content in the stroma were greater than those in the caterpillar body. The As content was substantially higher in the caterpillar body than it was in the stroma.

**Table 3:** Bioaccessibility of Cu, Pb, As, Cd and Hg in whole *C. sinensis*, stroma and caterpillar body

Part	Index (%)	Cu	Pb	As	Hg	Cd
	Total heavy metal content (mg kg^−1^)	15.21 ± 1.10	1.44 ± 0.46	9.70 ± 0.62	0.06 ± 0.02	0.11 ± 0.02
W	Bioaccessibility (%)	41.29 ± 1.86	40.11 ± 18.18	64.46 ± 6.68	18.91 ± 14.63	81.14 ± 10.66
	Gastric extract (%)	41.00 ± 2.00	11.25 ± 13.10	49.24 ± 3.95	10.09 ± 5.03	76.92 ± 28.31
	Intestinal extract (%)	0.29 ± 0.30	28.87 ± 12.63	15.21 ± 3.02	8.88 ± 9.58	9.53 ± 11.31
	Total heavy metal content (mg kg^−1^)	15.02 ± 0.95	1.39 ± 0.46	13.47 ± 0.84	0.06 ± 0.01	0.08 ± 0.01
C	Bioaccessibility (%)	48.26 ± 3.69	42.92 ± 5.14	66.15 ± 2.19	12.86 ± 3.57	87.07 ± 4.39
	Gastric extract (%)	44.19 ± 1.67	18.24 ± 11.00	52.54 ± 2.10	7.22 ± 1.88	83.94 ± 4.45
	Intestinal extract (%)	4.07 ± 4.06	24.68 ± 8.53	13.61 ± 1.63	5.70 ± 5.40	3.13 ± 0.23
	Total heavy metal content (mg kg^−1^)	18.63 ± 0.57	2.06 ± 0.44	1.49 ± 0.17	0.07 ± 0.01	0.22 ± 0.06
S	Bioaccessibility (%)	38.30 ± 3.80	30.53 ± 10.80	30.18 ± 9.62	7.46 ± 0.10	82.30 ± 0.49
	Gastric extract (%)	35.77 ± 0.90	13.68 ± 6.37	18.70 ± 5.70	3.05 ± 1.95	79.03 ± 0.28
	Intestinal extract (%)	2.53 ± 3.30	16.85 ± 4.49	11.45 ± 4.51	4.56 ± 2.19	3.28 ± 0.24

Results are expressed as mean ± SD, (n = 3)

*W* whole *C. sinensis*, *C* caterpillar body, *S* stroma

### Arsenic speciation analysis in whole *C. sinensis*, stroma and caterpillar body

In some parts of the samples, only As(III), As(V) and MMA were found, and MMA was only present in trace amounts (Table 4). The concentration of arsenic in *C. sinensis* was between 9.00 and 10.18 mg kg^−1^; almost all the dissolved arsenic is inorganic, making up approximately 8.69% of the total arsenic content. Among the extracted inorganic species, the concentrations of As(III) were 0.41–0.73, and the concentrations of As(V) were 0.25–0.35 mg kg^−1^. The As species that made up approximately 91.31% of the total arsenic were not identified. When the whole *C. sinensis* was divided into stroma and caterpillar body, the concentrations of As(III) were 0.07–0.08 mg kg^−1^ and were 0.39–0.50 mg kg^−1^ of As(V) in stroma, corresponding to a mean value of 34.20% the total arsenic in the stroma. The concentrations of As(III) were 0.48–0.64 and were 0.28–0.32 mg kg^−1^ of As(V) in caterpillar body. The mean value of these two inorganic arsenic forms made up 6.45% of the total arsenic content in the caterpillar body. Studies have shown that in the stroma of the *C. sinensis*, the As(V) content is greater than that of As(III). In contrast, As(III) content is greater than that of As(V) in caterpillar body.

**Table 4:** The content of arsenic species in the dilute nitric acid solution (HNO_3_: H_2_O = 2: 98) extract

Sample ID	Total As (mg kg^−1^)	Inorganic arsenic (mg kg^−1^)^a^			Organic arsenic (µg kg^−1^)				Unknown (mg kg^−1^)
		As(III)	As(V)	Inorganic arsenic extraction efficiency (%)	MMA	DMA	AsC	AsB	
W_1_	9.00 ± 0.21	0.41 ± 0.04	0.26 ± 0.01	7.45	N/A	ND	ND	ND	8.33
W_2_	9.93 ± 0.09	0.55 ± 0.02	0.35 ± 0.01	9.06	N/A	ND	ND	ND	9.03
W_3_	10.18 ± 0.15	0.73 ± 0.02	0.25 ± 0.01	9.56	N/A	ND	ND	ND	9.20
$\overline{w}$	9.70 ± 0.62	0.56 ± 0.16	0.29 ± 0.06	8.69	N/A	ND	ND	ND	8.85
S_1_	1.38 ± 0.14	0.08 ± 0.01	0.50 ± 0.01	41.80	N/A	ND	ND	ND	0.80
S_2_	1.41 ± 0.06	0.08 ± 0.00	0.39 ± 0.02	32.66	N/A	ND	ND	ND	0.95
S_3_	1.68 ± 0.08	0.07 ± 0.01	0.40 ± 0.02	28.14	N/A	ND	ND	ND	1.19
$\overline{\text{S}}$	1.49 ± 0.17	0.08 ± 0.01	0.43 ± 0.06	34.20	N/A	ND	ND	ND	0.98
C_1_	14.23 ± 0.10	0.59 ± 0.04	0.30 ± 0.01	6.30	N/A	ND	ND	ND	13.34
C_2_	13.62 ± 0.48	0.64 ± 0.07	0.28 ± 0.02	6.70	N/A	ND	ND	ND	12.71
C_3_	12.57 ± 0.03	0.48 ± 0.02	0.32 ± 0.00	6.35	N/A	ND	ND	ND	11.76
${\bar{\text{C}}}$	13.47 ± 0.84	0.57 ± 0.08	0.30 ± 0.02	6.45	N/A	ND	ND	ND	12.60

Results are expressed as mean ± SD, (n = 3)

N/A: trace amounts (LOD < N/A < LOQ); ND: not detected

^a^ The concentration calculated based on the original samples. $\bar{\varvec{x}}$: the average value

### As speciation analysis following in vitro gastrointestinal digestion

In different parts of *C. sinensis*, only As(III) and As(V) were detected in the in vitro digestion solution (Table 5). Inorganic arsenic was mainly dissolved in gastric juice; the average dissolved contents were 0.20 mg kg^−1^ in gastric juice and 0.02 mg kg^−1^ in intestinal juice. The inorganic arsenic in the in vitro digestion solution accounted for 2.33% of the total arsenic content; in which As(III) was accounted for 1.37% of the total and As(V) for 0.96%. The quantities of inorganic arsenic from the caterpillar body and stroma dissolved in the gastric juice and intestinal juice were 0.21 and 0.09 mg kg^−1^ and 0.02 and 0.02 mg kg^−1^, respectively, in which As(III) and As(V) accounted for 1.15 and 2.18% and 0.54 and 5.48%, respectively. And only parts of total As were dissolved. The results were consistent with the results in Table 4.

**Table 5:** The content of As species in the in vitro gastrointestinal digestion juice

Sample ID	Inorganic arsenic (mg·kg)^a^		Total As(III) (%)	Total As(V) (%)
	Gastric extract	Intestinal extract		
W_1_	0.22 ± 0.01	0.03 ± 0.00	1.55 ± 0.08	1.19 ± 0.04
W_2_	0.21 ± 0.01	0.01 ± 0.01	1.28 ± 0.04	0.97 ± 0.05
W_3_	0.19 ± 0.00	0.02 ± 0.00	1.29 ± 0.03	0.71 ± 0.01
$\overline{w}$	0.20 ± 0.02	0.02 ± 0.01	1.37 ± 0.15	0.96 ± 0.24
C1	0.30 ± 0.00	0.02 ± 0.00	1.53 ± 0.01	0.69 ± 0.01
C2	0.15 ± 0.05	0.01 ± 0.00	0.78 ± 0.25	0.43 ± 0.10
C3	0.19 ± 0.00	0.02 ± 0.00	1.15 ± 0.09	0.51 ± 0.06
$\overline{c}$	0.21 ± 0.08	0.02 ± 0.00	1.15 ± 0.38	0.54 ± 0.13
S1	0.09 ± 0.00	0.03 ± 0.02	3.04 ± 1.68	5.81 ± 0.04
S2	0.10 ± 0.00	0.02 ± 0.00	2.44 ± 0.19	6.02 ± 0.16
S3	0.09 ± 0.00	0.01 ± 0.01	1.07 ± 0.25	4.62 ± 1.03
$\overline{s}$	0.09 ± 0.01	0.02 ± 0.01	2.18 ± 1.00	5.48 ± 0.76

Results are expressed as mean ± SD, (n = 3)

^a^ The concentration calculated based on the original samples

### Estimation of health risks of heavy metals in *C. sinensis*

The DEs for Cu, Pb, As, Cd and Hg were 2.21, 0.21, 1.41, 0.02 and 0.01, respectively, which were all far below the corresponding tolerable limits set by the Joint FAO/WHO Expert Committee on Food Additives (JFCFA) (Table 6). The BTHQ values of these heavy metals were 0.0041, 0.0040, 0.5334, 0.0020 and 0.0005, respectively, all less than 1. The total BTHQ of the five heavy metals was 0.5440.

**Table 6:** Estimated dietary exposures (DE) and BTHQ values of Cu, Pb, As, Cd and Hg in *C. sinensis*

	Cu	Pb	As	Cd	Hg	Total
DE	2.21	0.21	1.41	0.02	0.01	3.86
BTHQ	0.0041	0.0040	0.5334	0.0020	0.0005	0.5440
PTDI	500	3.57	50	1	0.5	

DE is the dietary exposure (µg kg^−1^ body weight day^−1^)

PTDI is the provisional tolerable daily intake suggested by JECFA [28] given in µg kg^−1^ body weight day^−1^. The value of Hg refers to the limit of methyl mercury suggested by CAC

Based on the risk assessment results, As was the greatest contributor to heavy metal-related risks among the five elements, in agreement with previous findings. Other heavy metals in *C. sinensis* had no significant influence on human health.

## Discussion

### Bioaccessibility for heavy metal in *C. sinensis*

Bioaccessibility is defined as the proportion of a pollutant that can be dissolved in the gastrointestinal environment, indicating the relative amount of the pollutant in the matrix that can be absorbed by the body, and higher bioaccessibilities indicate greater potentials for absorbing pollutants. The heavy metals in Chinese herbal medicines enter the body through gastrointestinal tract, and cannot be 100% absorbed by digestive system. Therefore, the health risk of heavy metal could be overestimated if the total content of the heavy metal is used to carry out risk assessment instead of the total absorption content. Thus, bioaccessibility may be more accurate in assessing risks of heavy metals in Chinese herbal medicine.

Common methods for determining bioaccessibility of heavy metals are in vitro experiments; in this case, a simulated of gastrointestinal method was used for the simple method, short experimental period, and easily controlled experimental conditions. Dynamic in vitro gastrointestinal models mimic the gradual transit of ingested compounds through the digestive tract. In recent years, this method has been widely used to assess the risks of heavy metals in foods [20, 25, 26]. Studies have shown that the biochemical properties of heavy metals at various digestive stages are related to the characteristics of the heavy metals, their existent morphology, the type of matrix, and so on [27]. In this study, the bioaccessibility of heavy metals in *C. sinensis* and the morphology of arsenic in artificial gastrointestinal fluid were evaluated by in vitro experiments. The risks of heavy metals in *C. sinensis* were evaluated scientifically.

### As in vitro gastrointestinal digestion

Based on results of in vitro experiments, the bioaccessibility of arsenic in *C. sinensis* was 64.46%, indicating that arsenic in the *C. sinensis* could not be 100% dissolved by the artificial gastrointestinal fluid. And there were still a large number of unidentified arsenic species in the artificial gastrointestinal extract solution.

### The health risks of heavy metals in *C. sinensis*

*Cordyceps sinensis* is usually consumed for a long period of time to improve health. It is generally recommended to take *C. sinensis* for 2–3 months each year. The target hazard quotient (THQ) was used in this research, taking into consideration of daily dose and factors including most commonly consumed dosage forms and duration of consumption to evaluate potential and long-term risk of heavy metals in *C. sinensis* to human body. In the calculation of the THQ value, the duration of consumption was assumed to be 90 days, and the dosage was “the maximum dosage of 9 g recommended in Chinese Pharmacopoeia (2015 version)”. However, under actual conditions, the dosage is usually much less than 9 g because of the extremely high cost of *C. sinensis*, so the calculated THQ value is greater than its actual value and suggests an inflated risk. The experimental results suggested that there is no obvious risk from heavy metals in *C. sinensis* to human body. However, it is worth noting that the BTHQ value of arsenic is 0.5334 and the TBTHQ is 0.5440. If the required course of *C. sinensis* is longer than 90 days, the dose of *C. sinensis* needs to be reduced to avoid damage to the body from heavy metals.

### The standards of heavy metals in *C. sinensis*

Different medicinal materials grow in different environments with different life cycles and properties, causing variations in heavy metal contents. The unified standards of heavy metals in Chinese herbal medicines ignore some special properties of medicinal herbs. Chinese medicine is different from food in usages and dosages. Mode and frequency of drug use should be taken into consideration to get more equitable results of evaluation and standardization, when evaluating the risks of heavy metals and determining the standards for heavy metals in Chinese herbal medicines. This study showed that the heavy metals in *C. sinensis* present no obvious risks to human health in a reasonable taking way, indicating the excess of heavy metals in *C. sinensis* might be attributed to the unduly strict standard, and the heavy metal standards for *C. sinensis* need to loose properly.

## Conclusion

The five heavy metals cannot be 100% absorbed by human body. The content of arsenic in *C. sinensis* is high, but inorganic arsenic of strong toxic accounted for only 8.69% of the total in dilute nitric acid extracted solutions and for merely 2.33% of the total in the in vitro gastrointestinal solutions. The dietary exposure values of Cu, Pb, As, Cd and Hg were lower than the safety limits set by the Joint FAO/WHO Expert Committee on Food Additives (JFCFA) and the BTHQ of these heavy metals were all less than 1. The heavy metals in *C. sinensis* present no obvious risks to human health in a reasonable taking way. Perhaps, it is the time to loose the heavy metal standards for *C. sinensis* properly.

## Additional file

**Additional file 1.** The minimum standards of reporting checklist.
