# Supplementary material for: Bioaccessibility and risk assessment of heavy metals, and analysis of arsenic speciation in Cordyceps sinensis
Source: Chin Med. 2018 Jul 31;13:40. doi: 10.1186/s13020-018-0196-7 (PMC6069848; doi:10.1186/s13020-018-0196-7)
Supplement: Supplementary file 1 — Additional file 1. The minimum standards of reporting checklist. [file 13020_2018_196_MOESM1_ESM.docx]

# Minimum Standards of Reporting Checklist

*BioMed Central* advocates full and transparent reporting. Please ensure that your paper provides the information requested below where applicable. On submitting your paper you will be asked to confirm you have included this information, or give reasons for any instances where it is not made available. You will also be asked to upload this file and it should be cited in the Methods section.

## Experimental design and statistics

The following information should be included in the Methods section and inserted in the table below:

| **Question** | **Answer** |
| --- | --- |
| 1. The exact sample size (n) for each experimental group/condition (as a number, not a range). Include details of a power analysis if done, or any other relevant considerations that determined the choice of sample size. For n < 6, individual data values should be shown rather than summary statistics alone. | Three batches mixed samples of Cordyceps Sinensis were collected from Qinghai Province, and each batche has 30g. |
| 2. A description of sample collection that enables the reader to understand whether the samples represent technical or biological replicates, and an explanation of inclusion/exclusion criteria if samples or organisms were excluded from the analysis. | All samples were dried at 40℃ and stored at 4℃ before analysis. |
| 3. How samples/ organisms were allocated to experimental groups and processed, and full details of the randomisation procedure used (if relevant). | Each batch of *C. sinensis* samples was equally divided into two parts. One part, the whole *C. Sinensis* (W), was ground with a mortar and passed through a 50-mesh sieve and numbered W_1_, W_2_, and W_3_, respectively. The other part was separated into stroma (S) and caterpillar body (C), and those two portions were separately ground with a mortar and passed through a 50-mesh sieve and numbered S_1_, S_2_, S_3_ and C_1_, C_2_, C_3_, respectively. |
| 4. For sample assessment by human investigators, a statement on whether the investigator was blinded to group assignment and outcome assessment, and how this blinding was achieved and evaluated (if relevant). | Not applicable for my study. |
| 5. How many times each experiment shown was replicated and an indication of the extent of variation from experiment to experiment. | Each procedure was performed in triplicate. |
| 6. Information on the statistical methods and measures used. It should be clear whether the tests are one-sided or two-sided, whether there are adjustments for multiple comparisons, whether medians or means are being shown, whether error bars are standard deviations (SD), standard error of mean (SEM) or confidence intervals. | All statistical analysis were performed by SPSS 19.0 software (SPSS, IL, USA). |
| 7. A justification for the appropriateness of statistical tests used to assess significance. Do the data meet the assumptions of the tests? Is there an estimate of variation within each group of data, and is the variance similar between groups that are being statistically compared?  In addition, information essential to interpreting the data presented should be made available in the figure and table legends. If the study involves health interventions for human participants, please refer to the relevant reporting guidelines from the EQUATOR Network, and the Biosharing Portal for reporting checklists for biological and biomedical research, where applicable. | All statistical analysis were performed by SPSS 19.0 software (SPSS, IL, USA). |

## Research involving humans

If your research involved humans, please confirm you have adhered to the relevant reporting guideline from the [EQUATOR Network,](http://www.equator-network.org/) and included the completed checklist as an additional file with your submission:

|  |  | **Answer** (page and line number inserted/Not applicable for my study) |
| --- | --- | --- |
| - | I have followed the relevant reporting for my study type, and included a populated checklist with my submission | Not applicable for my study. |
| - | Not applicable for my study |  |

### Resources

A description of all resources used should be included in the Methods section, with enough information to allow them to be uniquely identified. The table below should be completed with confirmation that this was done (i.e. included in the Methods section) or is not applicable. If this has not been completed, but is applicable, you should contact the journal editorial staff before proceeding.

|  | **Answer** (page and line number inserted/Not applicable for my study) |
| --- | --- |
| •Antibodies: report source, catalogue code, characteristics, dilutions and how they were validated for the system under study. | Not applicable for my study. |
| •Cell lines: report source, whether identity has been authenticated and whether tested for mycoplasma contamination. We encourage researchers to check the NCBI database for contamination of cell lines. | Not applicable for my study. |
| •Organisms: report source, species, strain, sex, age, husbandry, inbred and strain characteristics of transgenic and mutant animals. | Not applicable for my study. |
| •Tools (software, databases and services): report standard tool name, provider and version number, if available. For antibodies, model organisms (mice, zebrafish and flies) and tools, authors are strongly encouraged to cite Research Resource Identifiers (RRIDs). To do so, please go to the Resource Identification Portal to search for your research resource and insert the reference text into your Methods section. | Not applicable for my study. |

### Availability of data and materials

The table below should be completed with confirmation that this was done (i.e.

included in the Methods section) or is not applicable.

|  | **Answer** (page and line number inserted/Not applicable for my study) |
| --- | --- |
| All datasets on which the conclusions of the paper rely must be either deposited in publicly available repositories (where available and ethically appropriate) or presented in the main paper or additional supporting files, in machine-readable format whenever possible. If authors are unable to fulfil this requirement, they should contact journal editorial staff, after checking our list of Recommended Repositories. | Not applicable for my study. |
| Links to deposited datasets, or datasets in additional files, should be explicitly referenced in a section entitled “Availability of Data and Materials”. Guidance on where to deposit your data can be found on the  Availability of Data and Materials policy page. | Not applicable for my study. |
| If computer code was used to generate results that are central to the paper’s conclusions, include a statement in the “Availability of data and materials” section to indicate how the code can be accessed. Include version information and any restrictions on availability. For deposited data and published code, a full reference with an accession number, doi or other unique identifier should be included in the reference list. | Not applicable for my study. |
| If reproducible materials are generated as a result of the research (for example new animal mutants), a statement on their availability should be included. | Not applicable for my study. |
